# Antiviral Activity of Isoquinolone Derivatives against Influenza Viruses and Their Cytotoxicity

**DOI:** 10.3390/ph14070650

**Published:** 2021-07-06

**Authors:** Yejin Jang, Jinhe Han, Xiaoli Li, Hyunjin Shin, Won-Jea Cho, Meehyein Kim

**Affiliations:** 1 Infectious Diseases Therapeutic Research Center, Korea Research Institute of Chemical Technology (KRICT), Daejeon 34114, Korea; erythro1@krict.re.kr (Y.J.); charliezz3656@gmail.com (H.S.); 2 College of Pharmacy, Chonnam National University, Gwangju 61186, Korea; hjh930116@gmail.com (J.H.); lixiaoli0425@gmail.com (X.L.); 3 Graduate School of New Drug Discovery and Development, Chungnam National University, Daejeon 34134, Korea

**Keywords:** antiviral, influenza virus, isoquinolone

## Abstract

Influenza viruses are one of the major causative agents for human respiratory infections. Currently, vaccines and antivirals approved for preventing and treating viral infections are available. However, limited protection efficacy and frequent emergence of drug-resistant viruses stand for a need for the development of antivirals with different chemical skeletons from existing drugs. Screening of a chemical library identified an isoquinolone compound (**1**) as a hit with 50% effective concentrations (EC_50_s) between 0.2 and 0.6 µM against the influenza A and B viruses. However, it exhibited severe cytotoxic effects with a 50% cytotoxic concentration (CC_50_) of 39.0 µM in canine kidney epithelial cells. To address this cytotoxic issue, we synthesized an additional 22 chemical derivatives. Through structure-activity, as well as structure-cytotoxicity relationship studies, we discovered compound **21** that has higher EC_50_ values ranging from 9.9 to 18.5 µM, but greatly alleviated cytotoxicity with a CC_50_ value over 300 µM. Mode-of-action and cell type-dependent antiviral experiments indicated that it targets viral polymerase activity and functions also in human cells. Here, we present a new class of viral polymerase inhibitors with a core skeleton of isoquinolone, of which antiviral activity could be better improved through following design and synthesis of its derivatives for drug development.

## 1. Introduction

Influenza A and B viruses, belonging to the family *Orthomyxoviridae,* are the main causative agents of human infectious respiratory diseases during seasonal epidemics and unpredictable pandemics [1]. The viral genome comprises eight negative-sense RNA segments that are coated with nucleoprotein (NP). The 5′- and 3′-termini of genomic RNA are bound to polymerase subunits composed of polymerase basic protein 2 (PB2), PB1, and polymerase acidic protein (PA), which are required for immediate initiation of RNA replication and transcription in the nucleus of an infected cell [2]. Currently, quadrivalent vaccines are available to prevent infection by H1N1 and H3N2 subtypes of influenza A virus, as well as by both the Victoria and Yamagata lineages of influenza B virus. However, protective efficacy of these vaccines is no greater than 50% per year on average [3]. Moreover, several months are required to produce a vaccine against newly emerging and re-emerging viruses that are less efficiently or not neutralized by the existing vaccines. Accordingly, as an alternative way to respond to the influenza outbreaks that lack vaccine preparedness, antiviral treatments play a pivotal role in preventing the spread of viral infections.

Three different classes of antivirals have been developed to treat influenza viruses; all comprise small molecules that target viral matrix protein 2 (M2), neuraminidase (NA), or viral polymerase complex [1,4]. Adamantanes, such as amantadine and rimantadine, are potent inhibitors that block the M2 protein of influenza A, but not influenza B, viruses. They are no longer recommended for clinical use due to the prevalence of drug-resistant viruses harboring single or multiple substitutions (S31N, L26I, and/or V27A) in M2. NA inhibitors, including oseltamivir, zanamivir, peramivir, and laninamivir, are licensed in many countries. Of these, oseltamivir phosphate, a pro-drug of oseltamivir carboxylate, is prescribed most globally. Recently circulating influenza A and B viruses remain sensitive to oseltamivir. However, the possible emergence of oseltamivir-resistant or less sensitive viruses harboring the H275Y mutation or additional substitutions in NA, as occurred in 2008, is a threat to public health through increased pathogenicity and transmissibility [5]. The last class of antivirals, favipiravir and baloxavir (formally known as baloxavir marboxil), affect the viral polymerase subunits PB1 (RNA-dependent RNA polymerase) and PA (cap-dependent endonuclease) activities, respectively [6,7]. Favipiravir has gained only limited approval for use in pandemic situations in Japan due to concerns about its potential embryotoxicity [8]. Studies of the clinical safety and efficacy of baloxavir show more promising results. Nevertheless, they have identified amino acid substitutions (mainly I38X in PA), which may reduce drug susceptibility [9]. These studies suggest that the influenza viral polymerase complex is an attractive target for drug development. However, we need to ensure the diversity of available polymerase inhibitors if we are to overcome the issues associated with drug resistance.

We found an isoquinolone compound (**1**) from a cell-based, high-throughput screening approach that shows inhibitor activity against both the influenza A and B viruses. The compound efficiently suppresses the viral RNA replication step, but has cytotoxic effects. To improve this limitation, we synthesized 22 chemical derivatives and identified a less toxic antiviral agent, compound **21**. Here, we show that compound **21** could act as the backbone for development of an antiviral drug inhibiting the influenza viral polymerase activity without exerting cytotoxic effects.

## 2. Results

### 2.1. Identification of Compound 1 with Anti-Influenza Viral Activity

**Figure 1:**
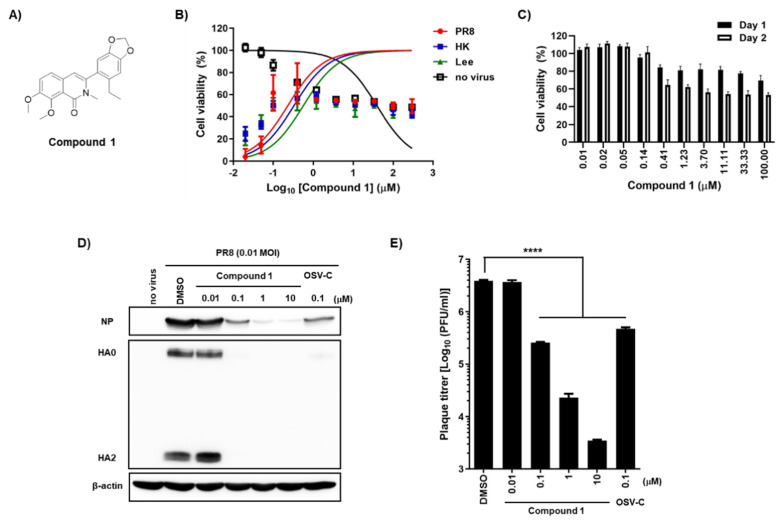
Antiviral activity of compound **1** against influenza A and B viruses and its toxicity in MDCK cells. (**A**) Chemical structure of compound **1**. (**B**) Antiviral activity and cytotoxicity of compound **1**. MDCK cells were mock-infected (no virus) or infected individually with A/Puerto Rico/8/34 (H1N1; PR8), A/Hong Kong/8/68 (H3N2; HK), or B/Lee/40 (Lee) in the presence of increasing concentrations of compound **1**. On day 2, cell viability was measured using fluorescein diacetate; the fluorescence intensity of mock-infected DMSO-treated cells and virus-infected DMSO-treated cells was set as 100% and 0%, respectively. (**C**) Comparison of the cytotoxicity of compound **1** on different days. The percentage viability of MDCK cells was measured in an MTT assay after treatment with increasing concentrations of compound **1** on days 1 (black bar) and 2 (white bar). (**D**) Inhibition of viral protein expression. MDCK cells were infected with PR8 for 1 h at a multiplicity of infection (MOI) of 0.01 and then treated with increasing concentrations of compound **1** or 0.1 µM OSV-C. On the next day, cell lysates were harvested for Western blot analysis using anti-NP and anti-HA2 antibodies. β-Actin was used as a loading control. Each protein is denoted on the left-handed side of the blots. (**E**) Plaque assay. Culture supernatants from samples prepared in (**D**) were serially diluted 10-fold and used to infect fresh MDCK cells. On day 3 post-infection, the number of plaque-forming units (PFU) was counted by crystal violet staining. In panels (**B**), (**C**), and (**E**), values are expressed as the means ± standard deviation from three different experiments. ****, *p* < 0.0001.

To secure compounds with anti-influenza activity, we screened a chemical library from the Korea Chemical Bank (Daejeon, Republic of Korea), which comprised 7000 different small molecules deposited by medicinal chemists. The fluorescent diacetate-based antiviral assay [10] identified 3-(6-ethylbenzo[*d*][1,3]dioxol-5-yl)-7,8-dimethoxy-2-methylisoquinolin-1(2*H*) (named compound **1**) as a hit compound, with 50% effective concentration (EC_50_) values ranging from 0.2 to 0.6 μM against both influenza A [A/Puerto Rico/8/34 (PR8) and A/Hong Kong/8/68 (HK)] and B/Lee/40 (Lee) viruses, and a 50% cytotoxic concentration (CC_50_) value of 39.0 μM in MDCK cells (Figure 1A,B; Table 1). Despite an excellent selective index (S.I.) of ≥65, incubation for 2 days led to >40% cell death at concentrations > 0.41 μM (Figure 1B). To exclude the possibility that the antiviral efficacy was mixed with cytotoxicity, it was needed to evaluate antiviral activity of compound **1** under subtoxic concentrations. To find this condition, we compared its cytotoxicity between days 1 and 2 using 3-(4,5-dimethylthiazol-2-yl)-2,5-diphenyl tetrazolium bromide (MTT) (Figure 1C). Consistent with the fluorescein diacetate assay (Figure 1B), the MTT assay revealed that a 2-day treatment of compound **1** at concentrations above 0.41 µM led to cell death more than 40%, while 1-day treatment below 11.1 µM resulted in a cell viability higher than 80%. On the basis of these subtoxic concentrations, both Western blot analysis and plaque titration assays were performed, showing that compound **1** suppressed influenza viral infection by reducing the expression of viral proteins, including NP and HA, in cells, as well as by reducing the number of viral progeny released into the culture supernatant (Figure 1D,E).

### 2.2. Compound 1 Inhibits Viral Polymerase Activity

**Figure 2:**
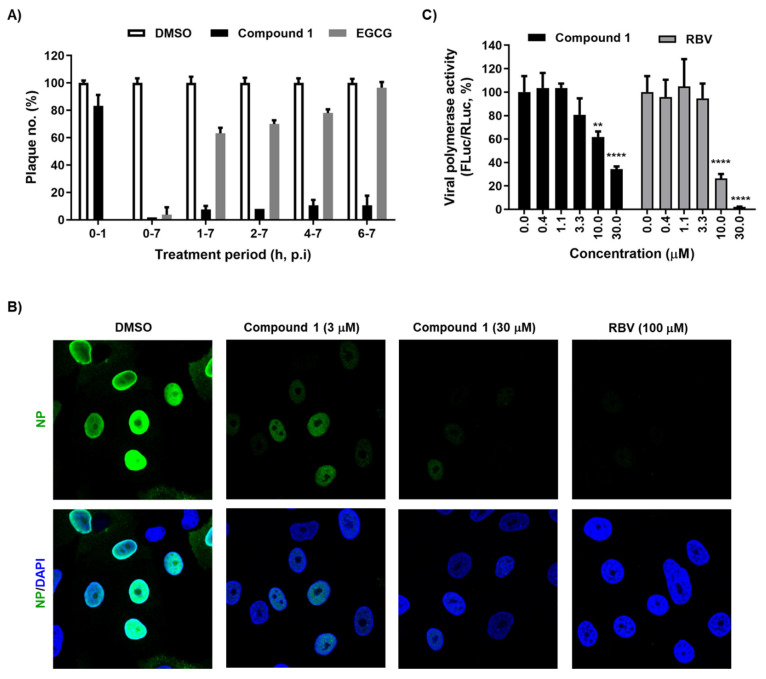
Inhibition of viral genome replication by compound **1**. (**A**) Time-of-addition experiment. MDCK cells in 48-well plates were infected with PR8 (MOI, 0.01) at 4 °C for 1 h. Unabsorbed virus was removed by washing with PBS. Compound **1** (10 µM) was added at different time points. EGCG (1 µM), which blocks virus entry, was used as a control. At 7 h post-infection, the supernatant was removed and overlay medium containing 0.6% CMC was overlaid for 3 days at 33 °C. Plaque numbers in DMSO-treated samples under each condition were set as 100%. (**B**) Confocal microscopy showing nuclear localization of NP. MDCK cells were infected with PR8 at an MOI of 3 in the presence of DMSO, compound **1** (3 or 30 µM), or ribavirin (RBV; 100 µM) at 33 °C for 5 h. Viral NP was visualized using an anti-NP antibody and Alexa 488-conjugated goat anti-mouse IgG (green), while cellular nuclei were counterstained with DAPI (blue). Original magnification, ×60. (**C**) Inhibition of viral polymerase activity. HeLa cells were transfected with plasmids expressing viral polymerase complex, including pVP-PB2, -PB1, -PA, or -NP (200 ng in well of a 24-well plate), together with reporter plasmids pHH21-FLuc (200 ng per well) and phRL-CMV (10 ng per well). At 6 h after transfection, cells were treated with increasing concentrations of compound **1** or RBV. On day 1, viral polymerase activity (%) was determined by measuring FLuc expression from pHH21-FLuc [normalized to *Renilla* luciferase (RLuc) expression from phRL-CMV]. In (**A**) and (**C**), values are expressed as the mean ± standard deviation from three different experiments. **, *p* < 0.01; ****, *p* < 0.0001.

Next, we performed a time-of-addition experiment to determine the step in the influenza virus life cycle targeted by compound **1**. Plaque titration assays revealed that exposure to compound **1** (10 μM) during viral adsorption (0–1 h) at 4 °C failed to induce antiviral activity, whereas EGCG, an entry inhibitor, blocked PR8 infection completely at the same condition (Figure 2A). However, treatment with compound **1** starting at any time point until 7 h post-infection consistently inhibited plaque formation by over 80%. This suggests that compound **1** could target virus penetration into cells or genome replication, rather than its attachment to cellular sialic acid receptors. To further evaluate this hypothesis, we compared the intracellular distribution of NP at 5 h post-infection in the presence or absence of compound **1**. Confocal microscopy showed few or no NP-positive cells when compound **1** was treated at concentrations of 3 or 30 μM (Figure 2B). To collect a more direct evidence, we adopted a cell-based reporter assay for influenza viral RdRp activity in which a negative strand of the firefly luciferase (FLuc) RNA transcript flanked with viral 5′ and 3′ UTRs is used as a template for synthesis of positive sense RNA, as well as mRNA, in the presence of viral polymerase complex. To address a concern that antiviral function observed could be induced limitedly in canine cells in a species-specific manner (Figure 1), a human cell line, HeLa, was applied to this polymerase assay. The results of the dual luciferase assay clarified that compound **1** suppressed viral RNA replication/transcription in a similar way to RBV (Figure 2C). Taken together, these mode-of-antiviral action experiments suggested that compound **1** works at the viral genome replication step.

### 2.3. Synthesis of Isoquinolone Derivatives

**Scheme 1:**
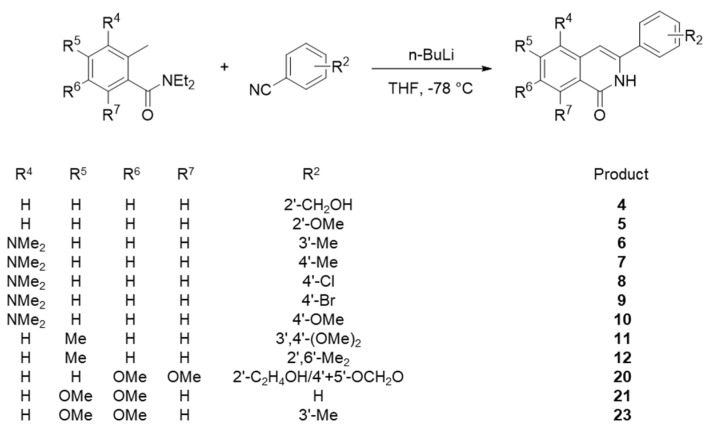
Synthesis of phenylisoquinolones **4**–**12**, **20**, **21**, and **23**.

Among 23 isoquinolone compounds, **1** and **17–19** have been synthesized according to our established protocol (Supplementary Scheme S1) [11,12]. Compounds **2**, **3**, **13–15** and **22** without the methylenedioxy group at R^2^ were separately synthesized (Supplementary Scheme S2) [13,14,15,16]. A newly designed synthetic method was used for phensylisoquinolone derivatives **7–10** and **21**, and these compounds were prepared in a one-step cyclization reaction using n-BuLi to link the N,N-diethyl-2-methylbenzamide and benzonitrile moieties (Scheme 1). These products were characterized by ^1^H NMR (Supplementary Figures S1 to S5). The remaining compounds, **4**, **5**, **6**, **11**, **12**, **20**, and **23**, were also synthesized according to Scheme 1 and our previous reports [15,16,17,18,19].

### 2.4. Structure-Activity Relationship: Isoquinolone Derivatives

As mentioned above, although compound **1** is a highly potent antiviral agent, it showed severe cytotoxic effects after treatment for 2 days (Figure 1B,C). Thus, we wondered which moiety within compound **1** was responsible for the cytotoxicity, and whether it could be improved by chemical modifications. To investigate the structure-activity relationship (SAR), as well as the structure-cytotoxicity relationship analyses, we chemically modified three rings (A, B, and C) of phenylisoquinolone, either independently or simultaneously (Table 1).

Taking the simplest compound with a basic skeleton, 3-phenylisoquinolone (**2)** insertion of substituents into Ring C revealed that a hydroxyl (**3**) or hydroxymethyl group (**4**) at the ortho position inhibited replication of the PR8, HK, and Lee influenza viral strains, with less cytotoxicity than compound **1**. The methoxy group (**5**) suppressed the Lee strain slightly, but was still cytotoxic. When the dimethylamino group was introduced at R^4^ in phenyl Ring C, the m-methyl derivative (**6**) was effective against Lee, but not against PR8 and HK. Interestingly, antiviral activity disappeared in derivatives with a para-substitution in Ring C (e.g., p-methyl (**7**), p-chloro (**8**), p-bromo (**9**), or p-methoxy (**10**) substitutions). Although none of these dimethylamino-substituted compounds showed cytotoxicity at the maximum concentration of 300 µM, additional dimethylamino derivatives were not considered for further modification and testing due to expectation of their loss of antiviral activities.

To investigate the influence of substitutions at R^5^ on cytotoxicity and antiviral activity, a methyl group was first introduced at that position, while R^2^ was modified. When dimethoxy moieties were substituted at the 3′ and 4′ positions of R^2^, the resulting compound **11** was non-toxic and showed considerable antiviral activity against the PR8 strain (S.I., >9.3), but its activity against the HK and Lee strains was weak (S.I., >3.2). Meanwhile, as shown for compound **12**, dimethyl modification at the 2′ and 6′ positions of the phenyl ring led to complete loss of antiviral activity. Bromination at R^3^ in compounds **11** and **12** generated **13** and **14**, respectively, which did not show significant antiviral activity. As another approach, derivatives methylated at R^1^ but not at R^5^ were synthesized, resulting in compounds **15**, **16**, and **17**. Compound **15** with a formyl group at the 2′ position showed neither antiviral activity nor severe toxicity. Even though its cytotoxicity was not fully reflected in the CC_50_ calculation, approximately 30–40% of cells were dead at concentrations > 100 µM (data not shown). Compound **16**, with a hydroxyl substitution at 2′ but a formyl group at R^3^, was not effective at subtoxic concentrations. Compound **17**, with a methoxy group at R^6^, was partially active against influenza A viral strains (EC_50_ values between 5.8 and 36.8 µM) but not against the influenza B virus. Notably, its cytotoxicity (CC_50_, 109.0 µM) was lower than that of compound **1** (CC_50_, 39.0 µM). Taken together, the results from active but less toxic compounds **2, 3**, **4**, **11**, and **17** suggest that the intrinsic antiviral activity of the hit compound **1** can be maintained with much lower cytotoxicity.

### 2.5. Structure-Activity Relationship: Dimethoxy Isoquinolone Derivatives

**Figure 3:**
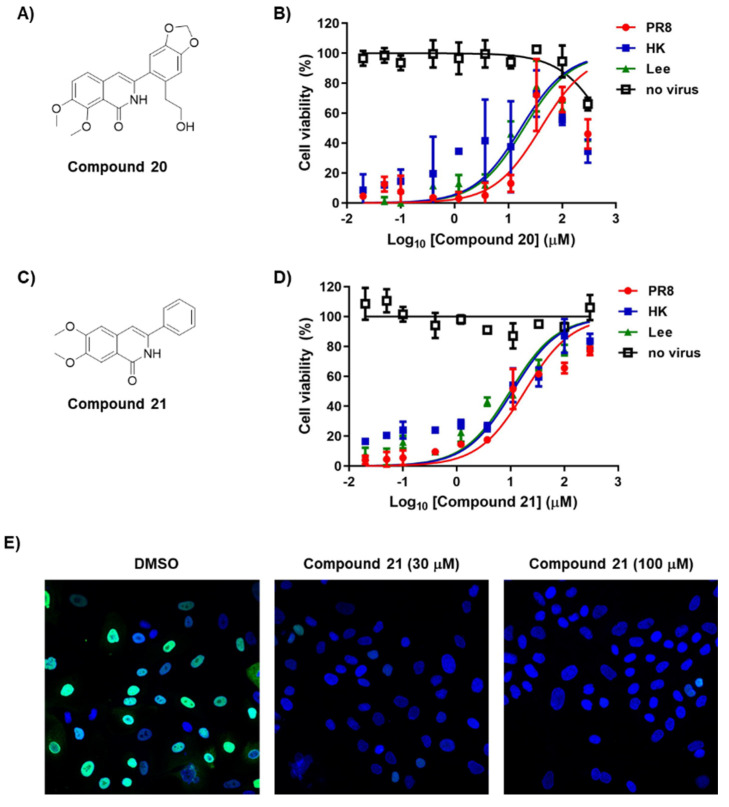
Antiviral activity and cytotoxicity of compounds **20** and **21**. (**A**,**C**) Chemical structure of compounds **20** and **21**. (**B**,**D**) Antiviral activity and cytotoxicity of compounds **20** and **21**. MDCK cells were mock-infected (no virus) or infected individually with influenza A viruses (PR8 and HK) or influenza B virus (Lee) in the presence of increasing concentrations of each compound. On day 2, percentage cell viability was measured using fluorescein diacetate; the fluorescence intensity of mock-infected DMSO-treated cells and virus-infected, DMSO-treated cells was set as 100% and 0%, respectively. (**E**) Confocal microscopy showing reduction of viral NP amounts by compound **21**. MDCK cells were infected with PR8 at an MOI of 3 in the presence of 0.2% DMSO or compound **21** (30 or 100 µM) at 33 °C for 5 h. Viral NP was visualized using an anti-NP antibody and Alexa 488-conjugated goat anti-mouse IgG (green), while cellular nuclei were counterstained with DAPI (blue). Original magnification, ×40.

Based on previous conjecture, we asked if it is possible to discover an active polymerase inhibitor (S.I., >15) without cytotoxicity at 300 µM, as observed in RBV. It was interesting that compound **17** exhibited considerable antiviral activity against the HK strain (S.I., 18.8) and was less cytotoxic than compound **1** (Table 1). From a comparison of their chemical structures between compounds **1** and **17**, we assumed that deleting the methoxy group at R^7^ would be associated directly with increased cell viability while maintaining antiviral activity. For an in depth SAR analysis, we focused on synthesizing additional dimethoxy derivatives of phenylisoquinolone, starting from compound **1** (Table 2). The ethyl group at the 2′ position of R^2^ in compound **1** was replaced by ethylene (**18**) and hydroxyethyl (**19**) groups. As expected, compound **18** had properties very similar to those of compound **1**, i.e., it was active but highly toxic, with a CC_50_ value of 40.3 µM. Surprisingly, compound **19** was not only active against all three strains, even with relatively high EC_50_ values ranging from 22.2 to 59.9 µM, but also highly improved the cytotoxicity profile (CC_50_, >300 µM). A comparative SAR analysis of **19** with **1** or with **18** indicated that the magnitude of R^7^-OMe-derived cytotoxicity depends on 2′ modification of R^2^. Notably, the demethylated derivative at R^1^ of non-toxic **19**, which generated compound **20**, increased the antiviral activity against the influenza A and B viruses by 2–3-fold. However, latent toxicity was still detected at the highest concentration, resulting in approximately 30% cell death at 300 µM (Figure 3A,B). It confirmed that being linked with a chemical entity at the 2′ position of R^2^, the methoxy group at R^7^ could be attributed to cytotoxicity.

We tried to synthesize more primitive phenylisoquinolone derivatives in which all side chains were removed except for the dimethoxyl groups at R^5^ and R^6^. Compound **21** exhibited ideal antiviral activity profiles, including a dose-dependent response, as well as tolerable cytotoxicity (Table 2; Figure 3C,D). It was active against all three viral strains tested, with EC_50_ values ranging from 9.9 to 18.5 µM, and with higher potency than a polymerase inhibitor, ribavirin (RBV). Most importantly, it was not cytotoxic, even at the highest concentration (300 µM). We evaluated its specific antiviral activity by visualizing the reduction of viral NP expression in PR8-infected cells using fluorescence microscopy (Figure 3E). By contrast, hydroxylation (**22**) or methylation (**23**) of the phenyl ring resulted in complete loss of activity (Table 2). This finding was intriguing because the 2′-OH of compound **3** hardly affected antiviral activity when compared with that of compound **2**. Collectively, it stresses again that the 2′ position in Ring C is involved in both antiviral activity and cytotoxicity, depending on the methoxylation stoichiometry of Ring A. In summary, the chemical modification of cytotoxic antiviral compound **1** identified seven safer (CC_50_, >300 µM) compounds (**2**–**4**, **11**, and **19**–**21**) exhibiting a broad spectrum antiviral activity. Among these, 6,7-dimethoxy-3-phenylisoquinolone (compound **21**) was most potent in the cell culture system, satisfying S.I. values over 15 against all three viral strains tested.

### 2.6. Anti-Influenza Viral Activity of Compound 21 in Human Cells by Suppressing Viral Polymerase Activity

**Figure 4:**
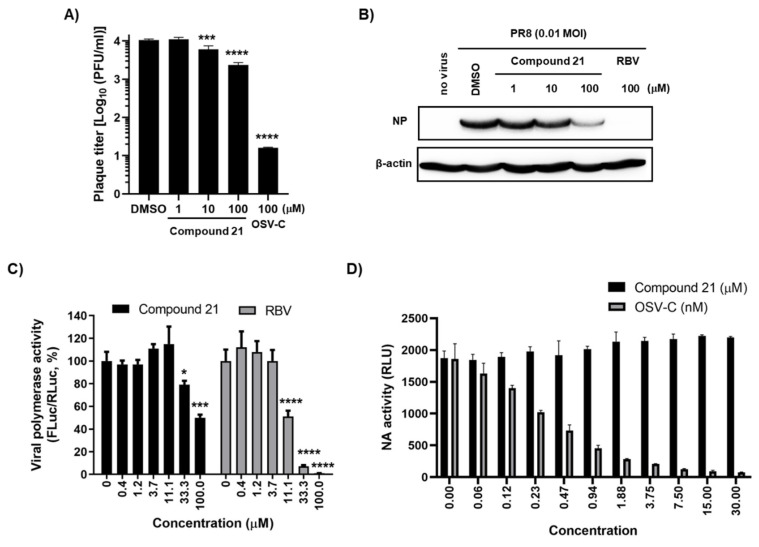
Antiviral activity of compound **21** in human cells. (**A**) Plaque assay. PR8-infected MDCK cells (MOI, 0.01) were treated with compound **21** or OSV-C, as a control, for one day. Culture supernatants were serially diluted 10-fold and used to infect fresh MDCK cells. On day 3, the number of plaques was counted by crystal violet staining. (**B**) Antiviral activity in A549 cells. Cells were infected with PR8 (MOI, 0.01) and treated with compound **21** or RBV, as a control. After SDS-PAGE and electrotransfer, viral NP protein was detected with anti-NP antibody and HRP-conjugated goat anti-rabbit IgG. β-Actin was used as a loading control. (**C**) Viral polymerase activity assay in HeLa cells. Cells were transfected with plasmids expressing viral polymerases, including pVP-PB2, -PB1, -PA, or -NP (200 ng in well of a 24-well plate), together with reporter plasmids pHH21-FLuc (200 ng per well) and phRL-CMV (10 ng per well). At 6 h after transfection, cells were treated with increasing concentrations of compound **1** or RBV. On day 1, viral polymerase activity (%) was determined by measuring FLuc normalized to RLuc. (**D**) NA activity assay. PR8 virus (2.8 × 10^3^ PFU) was pre-incubated with increasing concentrations of compound **21** or OSV-C at 37 °C for 20 min in 96-well plates. At 30 min after addition of a chemiluminescent NA substrate, luminescence was measured and expressed as relative luminescence units (RLU). In (**A**,**C**,**D**), values are expressed as the mean ± standard deviation from three different experiments. *, *p* < 0.05; ***, *p* < 0.001; ****, *p* < 0.0001.

A plaque assay confirmed that compound **21** inhibited progeny virus production into MDCK culture supernatants in a dose-dependent manner (Figure 4A). We wondered whether its antiviral efficacy can be reproducible in human cells. Human lung epithelial cells, A549, were infected with PR8 and treated with the compound for one day. Western blot analysis with the cell lysates revealed an inhibition of NP expression (Figure 4B). Furthermore, like the primary hit compound **1** (Figure 2C), it efficiently suppressed viral polymerase activity in HeLa cells (Figure 4C). However, our previous time-of-addition experiment with compound **1**, still showing antiviral activity between 6 and 7 h post-infection, motivatied us to investigate whether a later step of the virus life cycle can be influenced. As NA is a representative viral protein playing a pivotal role in the virus release, its activity was tested with a purified PR8 virus. The result showed that it had no inhibitory effect at a maximum concentration of 30 µM, while OSV-C efficiently blocked NA activity with a 50% inhibitory concentration (IC_50_) of 0.30 ± 0.04 nM (Figure 4D). These data clearly suggested that compound **21** affects viral polymerase activity, and its antiviral activity is reliable in human cells, ensuring its potential for the development of antivirals to treat human influenza infections through additional chemical modifications.

## 3. Discussion

Screening a chemical library identified that compound **1** had an inhibitory effect against both influenza A and B viruses with EC_50_ values ranging from 0.2 to 0.6 μM. It exhibited highly selective antiviral efficacy; nevertheless, it showed severe cytotoxicity (inducing > 40% cell death at concentrations > 0.41 μM on day 2). This was improved by reducing incubation time for 1 day, which resulted in 80% average viability at a concentration of 11.11 µM. However, the problem of intrinsic cytotoxicity had to be solved to make it more druggable. Therefore, we synthesized additional isoquinolone derivatives. Among the 22 modified compounds, we found that compound **21** did not affect cell viability when incubated with cells for 2 days at the maximum tested concentration of 300 µM. Although this modification reduced antiviral efficacy by 16.5–92.5-fold compared with compound **1**, compound **21** sufficiently suppressed influenza viral infection at subtoxic concentrations, showing a desirable antiviral profile with high S.I. values (>15) against all three viral strains tested (Figure 3D and Table 2). A time-of-addition study and enzymatic assay revealed that these isoquinolone compounds target the viral RNA replication step during the virus life cycle, not HA-mediated viral adsorption onto the cell surface or NA-mediated progeny viral release. It was pondered when compound **21** is a direct acting polymerase inhibitor, which viral protein among PB2, PB1 and PA could be a putative target molecule. To make rational conjecture, an in silico docking study was performed to compare the structural similarities and docking patterns by using the three polymerase inhibitors including VX787 (PB2 inhibitor), T705 (favipiravir, PB1 inhibitor) and Baloxavir (PA inhibitor) as controls. Interestingly, the modeling data displayed that compound **21** could interact with PB2 in a similar way to VX787 (Supplementary Figures S6–S8) [20,21,22]. In detail, compound **21** formed π-π stacked interactions with Phe104 and Phe23 of PB2 as observed in the binding pattern of VX787 to PB2. It also generated a hydrogen bond to Glu61 and H_2_O4140, which could increase its binding affinity to the protein. On the basis of the in silico docking analysis, it is expected that compound **21** could inhibit the polymerase activity by binding to PB2. However, at present, it is not yet experimentally determined whether it directly binds to one of the vRNP components, i.e., PB2, PB1, PA, or NP, or to cellular factors that are participated in viral genome amplification. Particularly, in the latter case, to address any concerns regarding the species-specific antiviral activity of the compounds which were discovered from MDCK cell-based screening, we evaluated its antiviral efficacy and polymerase inhibitory effect in human epithelial cells, A549 and HeLa, respectively (Figure 4B,C). The results ensured that even when it recognizes a host factor, it could not be a matter for antiviral drug development in further extensive chemical modifications. 

The structure-activity and structure-cytotoxicity relationship studies of isoquinolone derivatives had four outcomes: (i) the simplest core skeleton, phenylisoquinolone (**2**) and its 2′-substituted derivatives (**3** and **4**, but not **5**) are non-toxic but marginally active; (ii) complete loss of antiviral activity is induced by dimethylamino substitutions at R^4^ (as shown for **6** to **10**); (iii) methylation at R^1^ increases cytotoxicity (as shown for **1** and **16**–**18)**, although this can be moderated by substitution with either formyl (**15**) or hydroxyethanol (**19**) groups; and (iv) most of the compounds with mono-methoxy at R^6^ (**17**) or dimethoxy at both R^6^ and R^7^ (**18** to **20**) are effective but intrinsically toxic, while R^5^- and R^6^-substituted dimethoxy compounds are not toxic, but their antiviral activity is absolutely dependent on chemical substituents on R^2^ (**21** to **23**). Collectively, the data presented herein show that strategic chemical optimization of hit compound **1** generated compound **21**, which may be the basis for development of a novel therapeutic agent affecting the viral RNA replication step of different (sub)types of influenza viruses.

## 4. Materials and Methods

### 4.1. Chemical Synthesis

Isoquinolone derivatives were synthesized as described previously [11,12,13,14,15,16,17,18,19]. The structure of newly synthesized compounds **7–10** and **21** was confirmed by 300 MHz (Varian Unity Plus 300 MHz; Varian Inc., Palo Alto, CA, USA) and 400 MHz (Bruker Ascend 400 MHz; Bruker Daltonik, Bremen, Germany) nuclear magnetic resonance (NMR) spectrometry. Purity was estimated to be ≥99%.

### 4.2. Cells, Viruses, and Antiviral Compounds

Madin-Darby canine kidney (MDCK) cells, human cervical cancer HeLa cells and human lung epithelial A549 cells were purchased from the American Type Culture Collection (ATCC, Rockville, MD, USA) and cultured at 37 °C/5% CO_2_ in Minimum Essential Medium (MEM; Invitrogen, Carlsbad, CA, USA), Dulbecco’s Modified Eagle’s Medium (DMEM; Invitrogen) or Roswell Park Memorial Institute 1640 Medium (RPMI; Hyclone, Logan, UT, USA), respectively, supplemented with 10% FBS (Invitrogen). Influenza viruses A/Puerto Rico/8/34 (PR8), A/Hong Kong/8/68 (HK), and B/Lee/40 (Lee) were obtained from the ATCC. The two type A influenza strains were amplified in 10-day-old embryonated chicken eggs, while the type B strain was inoculated into MDCK cells in the presence of 2 μg/mL TPCK-treated trypsin at 37 °C for 3 days. The NA inhibitor, oseltamivir carboxylate (OSV-C; ≥98%), was purchased from United States Biological (Swampscott, MA, USA). The cell entry blocker (-)-epigallocatechin-3-gallate (EGCG, ≥95%) and the polymerase inhibitor ribavirin (RBV) were from Sigma-Aldrich (St. Louis, MO, USA). Stocks were prepared in water (for EGCG) or DMSO (for OSV-C and RBV); the final concentration of each was 50 mM.

### 4.3. Cytopathic Effect Assay

The fluorescein diacetate-based cell viability assay was performed as described previously [23]. Briefly, MDCK cells seeded on 96-well plates (3 × 10^4^ cells per well) were grown to confluency and then mock-infected or infected with the influenza A or B virus at a multiplicity of infection (MOI) of 0.0001. After viral absorption for 1 h, cells were washed with PBS and treated for 3 days at 33 °C with 3-fold serial dilutions of each compound in the presence of 2 μg/mL TPCK-treated trypsin (Sigma-Aldrich). Finally, they were incubated for 15 min at 37 °C with 30 μg/mL fluorescein diacetate. Cell viability was quantified using a Spectra Max M3 plate reader (Molecular Devices, Sunnyvale, CA) at an excitation wavelength of 485 nm and an emission wavelength of 538 nm. Both CC_50_ and EC_50_ values were determined by comparison with cell viability values from mock-infected and virus-infected cells, respectively, using GraphPad Prism 8.4.2 (GraphPad Software, La Jolla, CA, USA).

### 4.4. Immunoassay

For Western blot analysis, MDCK or A549 cells in 6-well plates (9 × 10^5^ cells per well) were infected with the PR8 virus at an MOI of 0.01 for 1 h and then treated with increasing concentrations of compound **1** or OSV-C in the presence of TPCK-trypsin (2 µg/mL for MDCK cells at 33 °C or 0.1 µg/mL for A549 cells at 35 °C). On the next day, cell lysates were subjected to 10% SDS-PAGE (30 µg per well), followed by electrotransfer to a PVDF membrane. Viral proteins NP and HA, and the cellular β-actin protein (a loading control) were detected using specific antibodies as described previously [24].

For fluorescence microscopy, PR8 virus-infected MDCK cells (MOI, 3) seeded onto 4-well slides (8.5 × 10^4^ cells per well) were treated with DMSO (delivery vehicle), compound **1**, or RBV at 33 °C for 5 h. After fixing and permeabilization, viral NP was visualized using an anti-NP antibody (Santa Cruz Biotechnology, Santa Cruz, CA, USA), followed by an Alexa 488-conjugated goat anti-mouse IgG (Invitrogen) [10]. Cells were counterstained with a Vectashield mounting medium containing DAPI (Vector Laboratories, Burlingame, CA, USA). Images were captured under a Zeiss LSM 700 confocal microscope (Carl Zeiss, Thornwood, NY, USA).

### 4.5. Plaque Titration

Cell culture supernatants harvested on day 1 post-viral infection in the presence or absence of compound **1** or OSV-C were centrifuged for 5 min at 3000 g and then serially diluted (10^−1^ to 10^−6^) in MEM. Fresh MDCK cells were seeded into 48-well plates (1 × 10^5^ cells per well) and incubated with the diluted samples for 1 h at 33 °C. Next, the cells were washed with PBS and incubated at 33 °C in the serum-free overlay medium [MEM containing 0.6% carboxymethyl cellulose (CMC; Sigma-Aldrich)] supplemented with 2 µg/mL TPCK-trypsin. On day 3, viral plaques were counted by staining with crystal violet.

In a time-of-addition experiment, the PR8 virus (MOI, 0.01) was loaded onto MDCK cells cultured in 48-well plates (1.5 × 10^5^ cells per well) for 1 h at 4 °C. After washing away unabsorbed virus, the cells were incubated at 33 °C for a further 7 h. In parallel with virus infection, 10 µM compound **1** or 1 µM EGCG (as a control for entry blocking) was added to infected cells during adsorption or at different times (0, 1, 2, 4, and 6 h) post-infection. At 7 h post-infection, all compounds were removed by washing twice with PBS prior to plaque titration as described above.

### 4.6. Viral Polymerase Assay

The influenza A viral polymerase activity assay was performed as described previously, with some modifications. Briefly, HeLa cells were seeded in 24-well plates (1 × 10^5^ cells per well). On the next day, the cells were transfected with plasmids expressing viral polymerases and NP (pVP-PB2, -PB2, -PA, or NP; 200 ng of each), together with reporter plasmids pHH21-FLuc (200 ng per well) and phRL-CMV (10 ng per well), which transcribe the negative-sense RNA of the FLuc gene flanked by UTRs from the influenza viral NS segment from RNA polymerase I and the RLuc mRNA from RNA polymerase II, respectively [10]. At 6 h post-transfection, the cells were treated with compound **1** or **21** or RBV. After 1 day, the cell lysates were harvested and tested in a dual luciferase assay (Promega, Madison, WI, USA).

### 4.7. NA Activity Assay

An NA activity assay was performed by using the NA-XTD Influenza Neuraminidase assay kit (Applied Biosystems, Foster City, CA, USA) [25,26]. The influenza A virus PR8 (2.8 × 10^3^ PFU) was mixed with the same volume of compounds at 37 °C for 20 min. After an addition of NA-XTD substrate, the mixture was incubated at room temperature for 30 min. The luminescent signal was maximized by the addition of an NA-XTD accelerator according to the manufacturer’s instructions and read using a microplate luminometer (Centro LB960 system; Berthold Technologies, Bad Wildbad, Germany). Half maximum inhibitory concentrations (IC_50_s) were determined using GraphPad Prism 8.4.2.

### 4.8. Statistical Analysis

Statistical analysis was performed using a two-tailed Student’s *t*-test. *p* values < 0.05 were considered significant. 

## Figures and Tables

**Table 1:** Antiviral activity of phenylisoquinolone derivatives against influenza viruses and toxicity in MDCK cells.

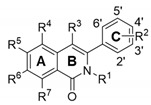											
Compound	Substitution							CC_50_ (µM) ^a^ against MDCK Cells	EC_50_ (µM) ^b^ against Influenza Virus (S.I. ^c^)		
	R^1^	R^2^	R^3^	R^4^	R^5^	R^6^	R^7^		PR8 ^d^	HK ^e^	Lee ^f^
**1**	Me	2′-Et, 4′+5′-OCH_2_O	H	H	H	OMe	OMe	39.0	0.2 (195.0)	0.3 (130.0)	0.6 (65.0)
**2**	H	H	H	H	H	H	H	>300.0	45.0 (>6.7)	73.4 (>4.1)	88.1 (>3.4)
**3**	H	2′-OH	H	H	H	H	H	>300.0	57.0 (>5.3)	103.4 (>2.9)	65.0 (>7.7)
**4**	H	2′-CH_2_OH	H	H	H	H	H	>300.0	52.4 (>5.7)	81.3 (>3.7)	17.2 (>17.4)
**5**	H	2′-OMe	H	H	H	H	H	120.4	>120.4 (n.d. ^g^)	>120.4 (n.d.)	1.4 (86.0)
**6**	H	3′-Me	H	NMe_2_	H	H	H	>300.0	>300.0 (n.d.)	>300.0 (n.d.)	58.7 (>5.1)
**7**	H	4′-Me	H	NMe_2_	H	H	H	>300.0	>300.0 (n.d.)	>300.0(n.d.)	>300.0 (n.d.)
**8**	H	4′-Cl	H	NMe_2_	H	H	H	>300.0	>300.0 (n.d.)	>300.0 (n.d.)	>300.0 (n.d.)
**9**	H	4′-Br	H	NMe_2_	H	H	H	>300.0	>300.0 (n.d.)	>300.0 (n.d.)	>300.0 (n.d.)
**10**	H	4′-OMe	H	NMe_2_	H	H	H	>300.0	>300.0 (n.d.)	>300.0 (n.d.)	>300.0 (n.d.)
**11**	H	3′,4′-(OMe)_2_	H	H	Me	H	H	>300.0	32.3 (>9.3)	94.5 (>3.2)	74.8 (>4.0)
**12**	H	2′,6′-Me_2_	H	H	Me	H	H	>300.0	>300.0 (n.d.)	>300.0 (n.d.)	>300.0 (n.d.)
**13**	H	3′,4′-(OMe)_2_	Br	H	Me	H	H	>300.0	177.0 (>1.7)	172.5 (>1.7)	>300.0 (n.d.)
**14**	H	2′,6′-Me_2_	Br	H	Me	H	H	>300.0	>300.0 (n.d.)	>300.0 (n.d.)	>300.0 (n.d.)
**15**	Me	2′-CHO	H	H	H	H	H	>300.0	>300.0 (n.d.)	>300.0 (n.d.)	>300.0 (n.d.)
**16**	Me	2′-OH	CHO	H	H	H	H	148.5	>148.5 (n.d.)	>148.5 (n.d.)	>148.5 (n.d.)
**17**	Me	2′-Et, 4′+5′-OCH_2_O	H	H	H	OMe	OH	109.0	36.8 (3.0)	5.8 (18.8)	>109.0 (n.d.)
**AMT ^h^**								>300.0	>300.0 (n.d.)	1.6 (>187.5)	>300.0 (n.d.)
**RBV ^i^**								>300.0	25.0 (>16.2)	43.8 (>19.7)	48.4 (>30.6)
**OSV-C ^j^**								>300.0	0.05 (>6000)	0.03 (>10,000)	0.37 (>810.8)

^a^ 50% cytotoxic concentration, concentration at which cell viability was reduced by 50%; ^b^ 50% effective concentration, concentration that is required to improve the viability of influenza virus-infected MDCK cells by 50%; ^c^ Selectivity index, the ratio of CC_50_/EC_50_; ^d^ A/Puerto Rico/8/34 (H1N1); ^e^ A/Hong Kong/8/68 (H3N2); ^f^ B/Lee/40; ^g^ not determined; ^h^ amantadine hydrochloride; ^i^ ribavirin; ^j^ oseltamivir carboxylate.

**Table 2:** Antiviral activity of dimethoxyphenylisoquinolone derivatives against influenza viruses and toxicity in MDCK cells.

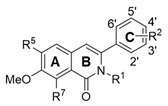									
Compound	Substitution					CC_50_ (µM) ^a^ against MDCK Cells	EC_50_ (µM) ^b^ against Influenza Virus (S.I. ^c^)		
	R^1^	R^2^	R^5^	R^6^	R^7^		PR8 ^d^	HK ^e^	Lee ^f^
**1**	Me	2′-Et, 4′+5′-OCH_2_O	H	OMe	OMe	39.0	0.2 (195.0)	0.3 (130.0)	0.6 (65.0)
**18**	Me	2′-CHCH_2_, 4′+5′-OCH_2_O	H	OMe	OMe	40.3	2.8 (14.4)	1.4 (28.8)	1.1 (36.6)
**19**	Me	2′-C_2_H_4_OH, 4′+5′-OCH_2_O	H	OMe	OMe	>300.0	59.9 (>5.0)	46.2 (>6.5)	22.2 (>13.5)
**20**	H	2′-C_2_H_4_OH, 4′+5′-OCH_2_O	H	OMe	OMe	>300.0	29.8 (>10.1)	11.9 (>25.2)	15.6 (>19.2)
**21**	H	H	OMe	OMe	H	>300.0	18.5 (>16.2)	10.9 (>27.5)	9.9 (>30.3)
**22**	H	2′-OH	OMe	OMe	H	>300.0	>300.0 (n.d. ^g^)	>300.0 (n.d.)	>300.0 (n.d.)
**23**	H	3′-Me	OMe	OMe	H	>300.0	>300.0 (n.d.)	>300.0 (n.d.)	>300.0 (n.d.)
**AMT ^h^**						>300.0	>300.0 (n.d.)	1.6 (>187.5)	>300.0 (n.d.)
**RBV ^i^**						>300.0	25.0 (>16.2)	43.8 (>19.7)	48.4 (>30.6)
**OSV-C ^j^**						>300.0	0.05 (>6000)	0.03 (>10,000)	0.37 (>810.8)

^a^ 50% cytotoxic concentration, concentration at which cell viability was reduced by 50%; ^b^ 50% effective concentration, concentration required to improve the viability of influenza virus-infected MDCK cells by 50%; ^c^ Selectivity index, the ratio of CC_50_/EC_50_; ^d^ A/Puerto Rico/8/34 (H1N1); ^e^ A/Hong Kong/8/68 (H3N2); ^f^ B/Lee/40; ^g^ not determined; ^h^ amantadine hydrochloride; ^i^ ribavirin; ^j^ oseltamivir carboxylate.

## Data Availability

Data is contained within the article and Supplementary Material.

## Supplementary Materials

The following are available online at https://www.mdpi.com/article/10.3390/ph14070650/s1, Scheme S1: Synthesis of phenylisoquinolones **1** and **17**–**19**; Scheme S2: Synthesis of phenylisoquinolones **2**, **3**, **13**–**15** and **22**; Scheme S3: Synthesis of phenylisoquinolone **16**; Figure S1: 1H NMR (400 MHz, DMSO-d6) spectrum of **7**; Figure S2: 1H NMR (400 MHz, DMSO-d6) spectrum of **8**; Figure S3: 1H NMR (400 MHz, DMSO-d6) spectrum of **9**; Figure S4: 1H NMR (400 MHz, DMSO-d6) spectrum of **10**; Figure S5: 1H NMR (400 MHz, DMSO-d6) spectrum of **21**; Figure S6. Docking pattern comparison of VX787 (A and B) and compound **21** (C and D) to PB2; Figure S7: Docking pattern comparison of T705 (A and B) and compound **21** (C and D) to PB1; Figure S8. Docking pattern comparison of Baloxavir (A and B) and compound **21** (C and D) to PA.
