# Supplementary material for: Antiviral Activity of Isoquinolone Derivatives against Influenza Viruses and Their Cytotoxicity
Source: Pharmaceuticals (Basel). 2021 Jul 6;14(7):650. doi: 10.3390/ph14070650 (PMC8308780; doi:10.3390/ph14070650)
Supplement: Supplementary file 1 [file pharmaceuticals-14-00650-s001.zip › Supplementary data (Jang) R2C1 Corrected.pdf]

Supplementary Materials

Antiviral activity of isoquinolone derivatives against influenza viruses and their cytotoxicity

Yejin Jang [1,†], Jinhe Han [2,†], Xiaoli Li [2], Hyunjin Shin [1], Won-Jea Cho [2,*] and Meehyein Kim [1,3,*]

[1] Infectious Diseases Therapeutic Research Center, Korea Research Institute of Chemical Technology (KRICT), Daejeon 34114, Republic of Korea; erythro1@krict.re.kr (Y.J.), charliezz3656@gmail.com (H.S.), mkim@krict.re.kr (M.K.)

[2] College of Pharmacy, Chonnam National University, Gwangju, 61186, Republic of Korea; hjh930116@gmail.com (J.H.), lixiaoli0425@gmail.com (X.L.), wjcho@jnu.ac.kr (W.-J. Cho)

[3] Graduate School of New Drug Discovery and Development, Chungnam National University, Daejeon 34134, Republic of Korea

Table of Contents

Contents	Page number
**Supplementary Scheme S1.** Synthesis of phenylisoquinolones **1** and **17-19**<br>**Supplementary Scheme S2.** Synthesis of phenylisoquinolones **2**, **3**, **13-15** and **22**<br>**Supplementary Scheme S3.** Synthesis of phenylisoquinolone **16**	S2-S4
**Experimental data of newly synthesized compounds**	S5-S6
**References**	S7
**Supplementary Figure S1.** ^{1}H NMR (400 MHz, DMSO-d6) spectrum of **7**<br>**Supplementary Figure S2.** ^{1}H NMR (400 MHz, DMSO-d6) spectrum of **8**<br>**Supplementary Figure S3.** ^{1}H NMR (400 MHz, DMSO-d6) spectrum of **9**<br>**Supplementary Figure S4.** ^{1}H NMR (400 MHz, DMSO-d6) spectrum of **10**<br>**Supplementary Figure S5.** ^{1}H NMR (400 MHz, DMSO-d6) spectrum of **21**<br>**Supplementary Figure S6.** Docking pattern comparison of VX787 (A and B) and compound **21** (C and D) to PB2<br>**Supplementary Figure S7.** Docking pattern comparison of T705 (A and B) and compound **21** (C and D) to PB1<br>**Supplementary Figure S8.** Docking pattern comparison of Baloxavir (A and B) and compound **21** (C and D) to PA	S8-S15

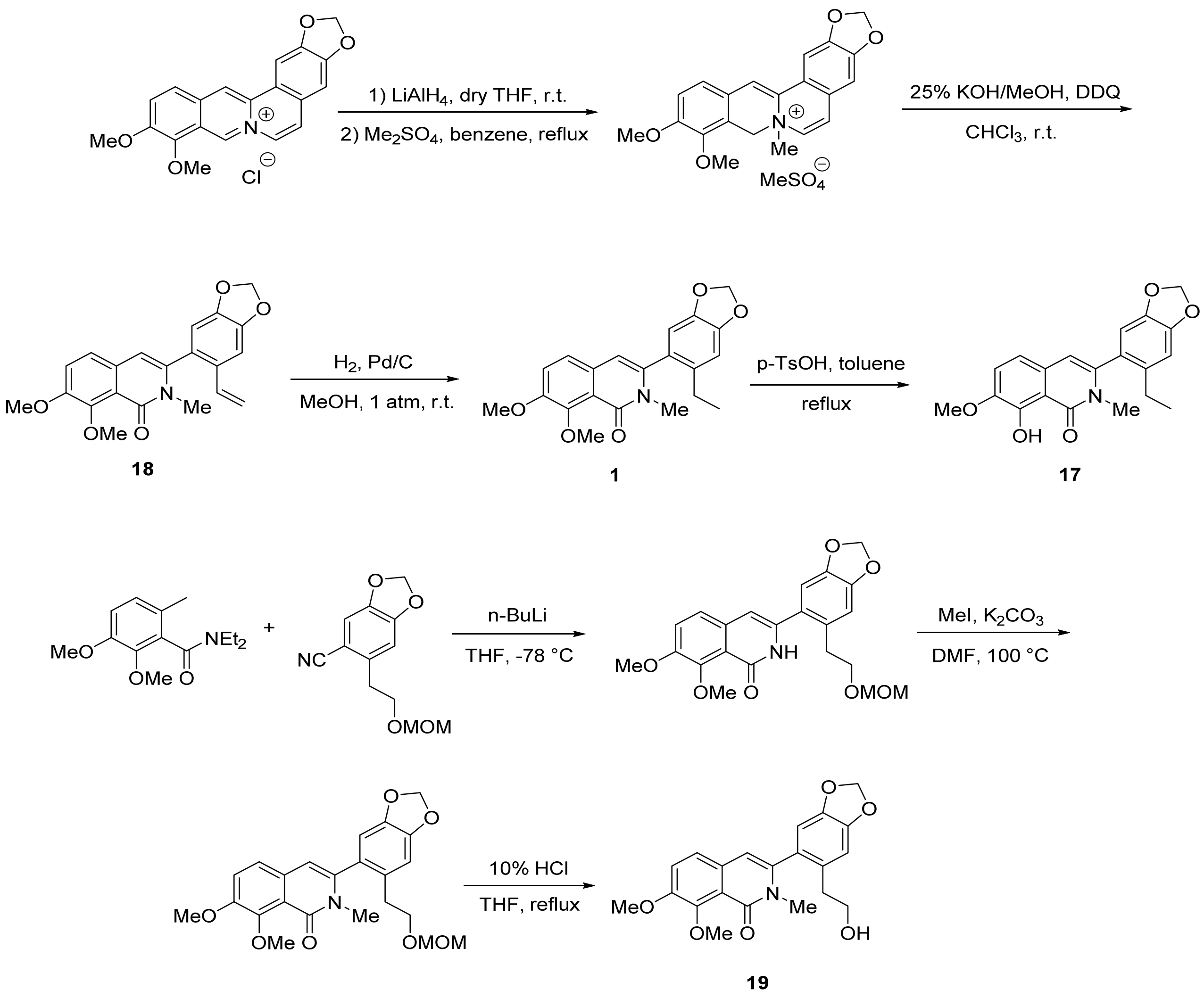

Supplementary Scheme S1. Synthesis of phenylisoquinolones **1** and **17-19** [1, 2].

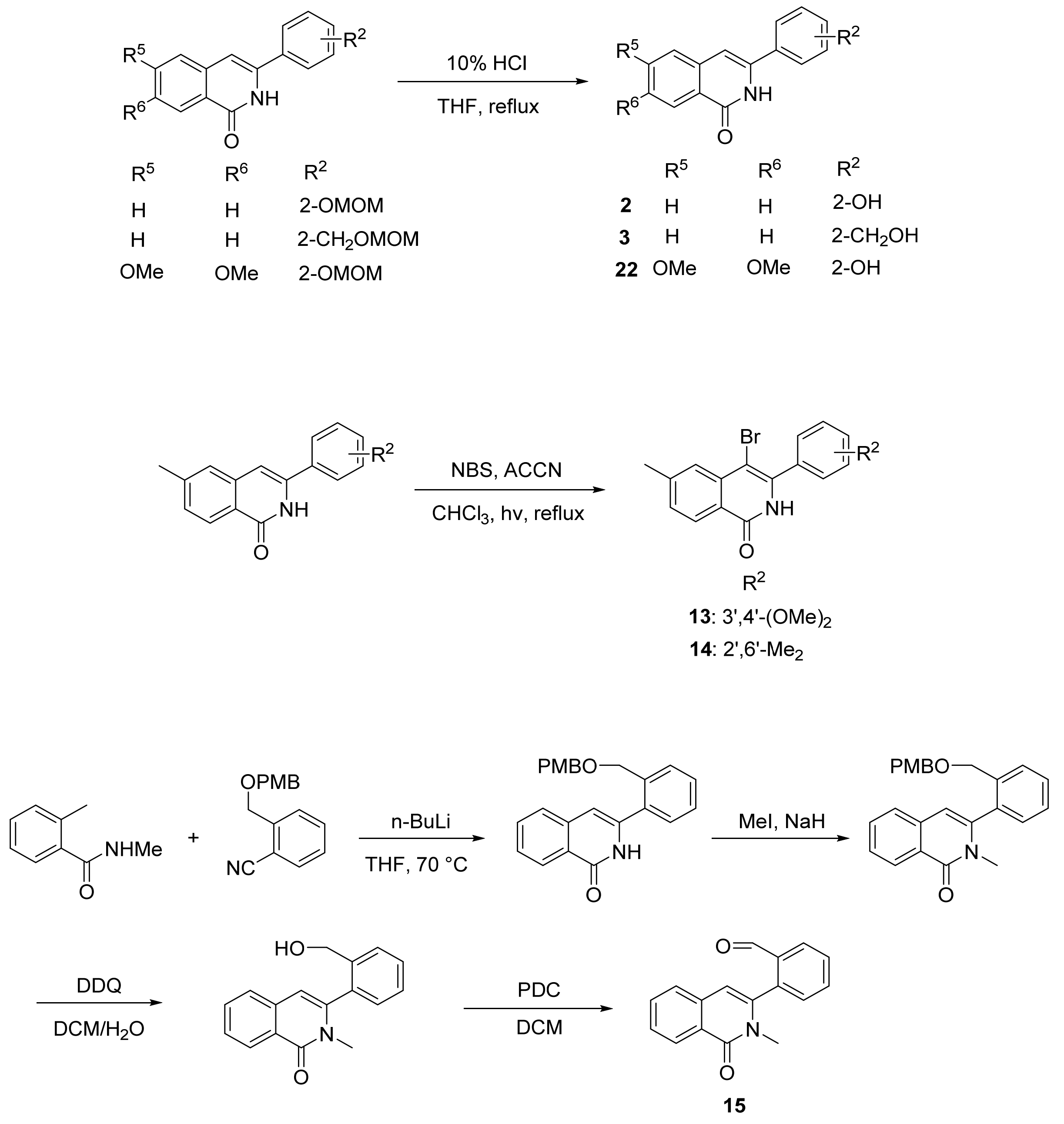

Supplementary Scheme S2. Synthesis of phenylisoquinolones **2**, **3**, **13-15** and **22** [3-6].

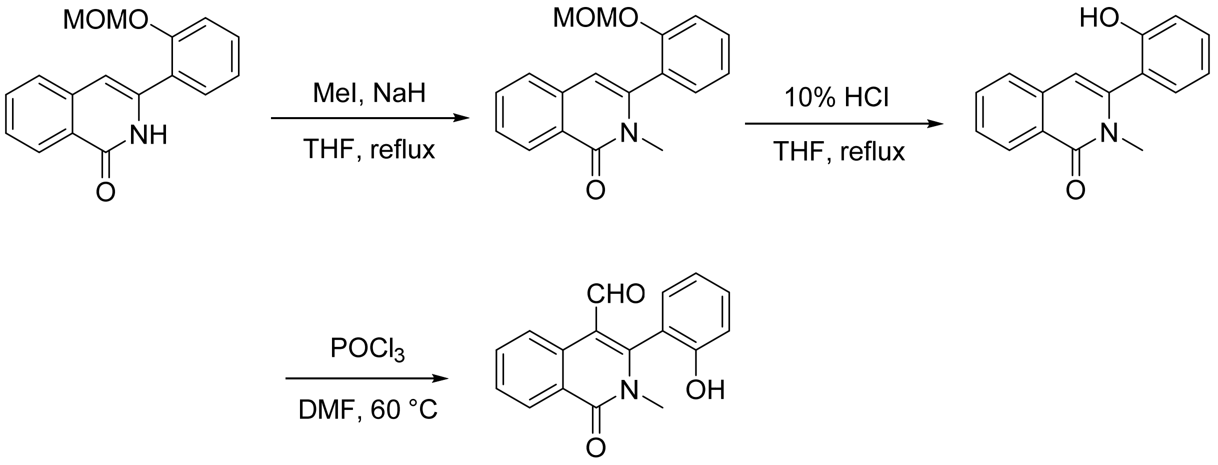

16

Supplementary Scheme S3. Synthesis of phenylisoquinolone **16** [7].

Experimental data of newly synthesized compounds

*3-(4-Chlorophenyl)-5-(dimethylamino)isoquinolin-1(2H)-one (**07**)*

The procedures described for **06** were used with 2.5 M *n*-BuLi in hexane (7.8 mL, 19.6 mmol), 3-(dimethylamino)-N,N-diethyl-2-methylbenzamide (2 g, 9.8 mmol), 4-chlorobenzonitrile (1.7 g, 11.8 mmol) and dry THF to obtain **07** (640 mg, 21%). ^{1}H-NMR (300 MHz, $CDCl_3$) δ: 11.5 (s, 1H), 8.00–7.50 (m, 7H), 6.92 (s, 1H), 2.78 (s, 6H).

*3-(4-Bromophenyl)-5-(dimethylamino)isoquinolin-1(2H)-one (**08**)*

The procedures described for **06** were used with 2.5 M *n*-BuLi in hexane (7.7 mL, 19.5 mmol), 3-(dimethylamino)-N,N-diethyl-2-methylbenzamide (2 g, 9.74 mmol), 4-bromobenzonitrile (2.2 g, 11.7 mmol) and dry THF to obtain **08** (725 mg, 21%). ^{1}H-NMR (300 MHz, $CDCl_3$) δ: 10.87 (s, 1H), 8.08 (d, J = 7.5 Hz, 1H), 7.79–7.25 (m, 6H), 7.16 (s, 1H), 2.86 (s, 6H).

*5-(Dimethylamino)-3-(4-methoxyphenyl)isoquinolin-1(2H)-one (**09**)*

The procedures described for **06** were used with 2.5 M *n*-BuLi in hexane (7.8 mL, 19.4 mmol), 3-(dimethylamino)-N,N-diethyl-2-methylbenzamide (2.0 g, 9.7 mmol), 4-methoxybenzonitrile (1.6 g, 11.7 mmol) and dry THF to obtain **09** (640 mg, 22%). ^{1}H-NMR (300 MHz, $CDCl_3$) δ: 10.30 (s, 1H), 8.03 (d, J = 7.0Hz, 1H), 7.95–7.2 (m, 6H), 7.0 (s, 1H), 3.88 (s, 3H), 2.85 (s, 6H).

*3-(3-Methoxyphenyl)-5-methylisoquinolin-1(2H)-one (**10**)*

The procedures described for **06** were used with *N*,*N*-diethyl-2,3-dimethylbenzamide (1.02 g, 5 mmol), 3-methoxybenzonitrile (832 mg, 6.25 mmol), 2.5 M *n*-BuLi in hexane (5 mL, 12 mmol) and dry THF to obtain **10** (1 g, 75%). ^{1}H-NMR (400 MHz, $CDCl_3$) δ: 10.42 (s, 1H), 8.28 (d, *J* = 8.0, 1H), 7.51 (d, *J* = 7.2 Hz, 1H), 7.45–7.41 (m, 1H), 7.39–7.31 (m, 3H), 7.03–7.00 (m, 1H), 6.89 (s, 1H), 3.92 (s, 3H), 2.59 (s, 3H).

*6,7-Dimethoxy-3-phenylisoquinolin-1(2H)-one (**21**)*

The procedures described for **06** were used with 3-(dimethylamino)-N,N-diethyl-2-methylbenzamide (1 g, 3.98 mmol), benzonitrile (468 mg, 4.53 mmol), 2.5 M *n*-BuLi in hexane (3.2 mL, 7.96 mmol) and dry THF to obtain **21** (98 mg, 8%). ^{1}H-NMR (400 MHz, $CDCl_3$) δ: 9.06 (s, 1H), 7.78 (s, 1H), 7.65–7.62 (m, 2H), 7.53–7.43 (m, 3H), 6.97 (s, 1H), 6.71 (s, 1H), 4.03 (s, 3H), 4.02 (s, 3H).

References

[1] T.N. Le, S.G. Gang, W.J. Cho, A versatile total synthesis of benzo[c]phenanthridine and protoberberine alkaloids using lithiated toluamide-benzonitrile cycloaddition, J Org Chem, 69 (2004) 2768-2772.

[2] W.J. Cho, S.J. Yoo, M.J. Park, B.H. Chung, C.O. Lee, Synthesis and antitumor activity of 3-arylisoquinoline derivatives, Arch Pharm Res, 20 (1997) 264-268.

[3] Y. Jin, D.B. Khadka, S.H. Yang, C. Zhao, W.-J. Cho, Synthesis of novel 5-oxaprotoberberines as bioisosteres of protoberberines, Tetrahedron Letters, 55 (2014) 1366-1369.

[4] H.T. Van, Q.M. Le, K.Y. Lee, E.S. Lee, Y. Kwon, T.S. Kim, T.N. Le, S.H. Lee, W.J. Cho, Convenient synthesis of indeno[1,2-c]isoquinolines as constrained forms of 3-arylisoquinolines and docking study of a topoisomerase I inhibitor into DNA-topoisomerase I complex, Bioorg Med Chem Lett, 17 (2007) 5763-5767.

[5] D.B. Khadka, H. Woo, S.H. Yang, C. Zhao, Y. Jin, T.N. Le, Y. Kwon, W.J. Cho, Modification of 3-arylisoquinolines into 3,4-diarylisoquinolines and assessment of their cytotoxicity and topoisomerase inhibition, Eur J Med Chem, 92 (2015) 583-607.

[6] H.T. Van, W.J. Cho, Structural modification of 3-arylisoquinolines to isoindolo[2,1-b]isoquinolinones for the development of novel topoisomerase 1 inhibitors with molecular docking study, Bioorg Med Chem Lett, 19 (2009) 2551-2554.

[7] D.B. Khadka, S.H. Yang, S.H. Cho, C. Zhao, W.-J. Cho, Synthesis of 12-oxobenzo[c]phenanthridinones and 4-substituted 3-arylisoquinolones via Vilsmeier–Haack reaction, Tetrahedron, 68 (2012) 250-261.

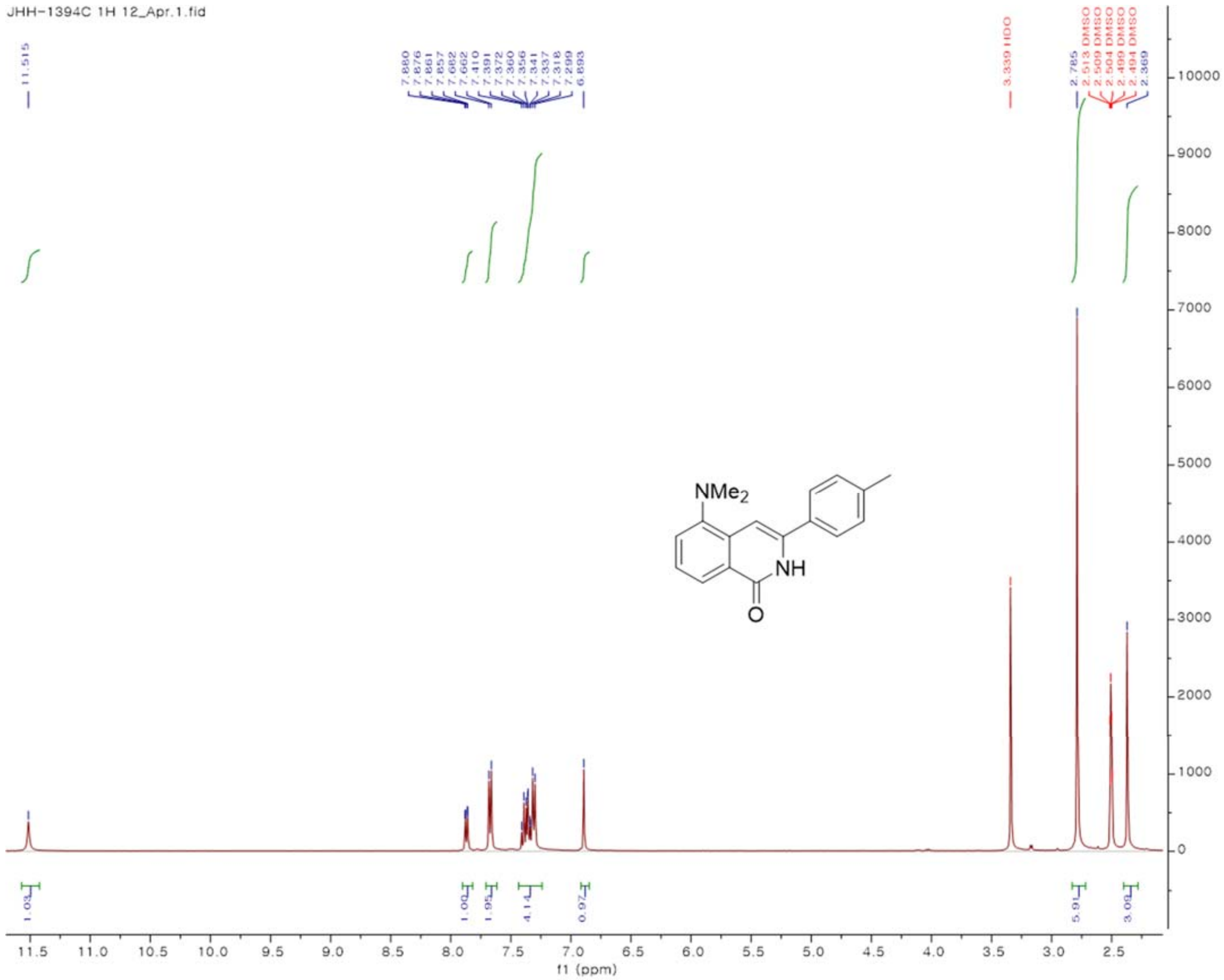

Supplementary Figure S1. ^{1}H NMR (400 MHz, DMSO-d6) spectrum of **7**.

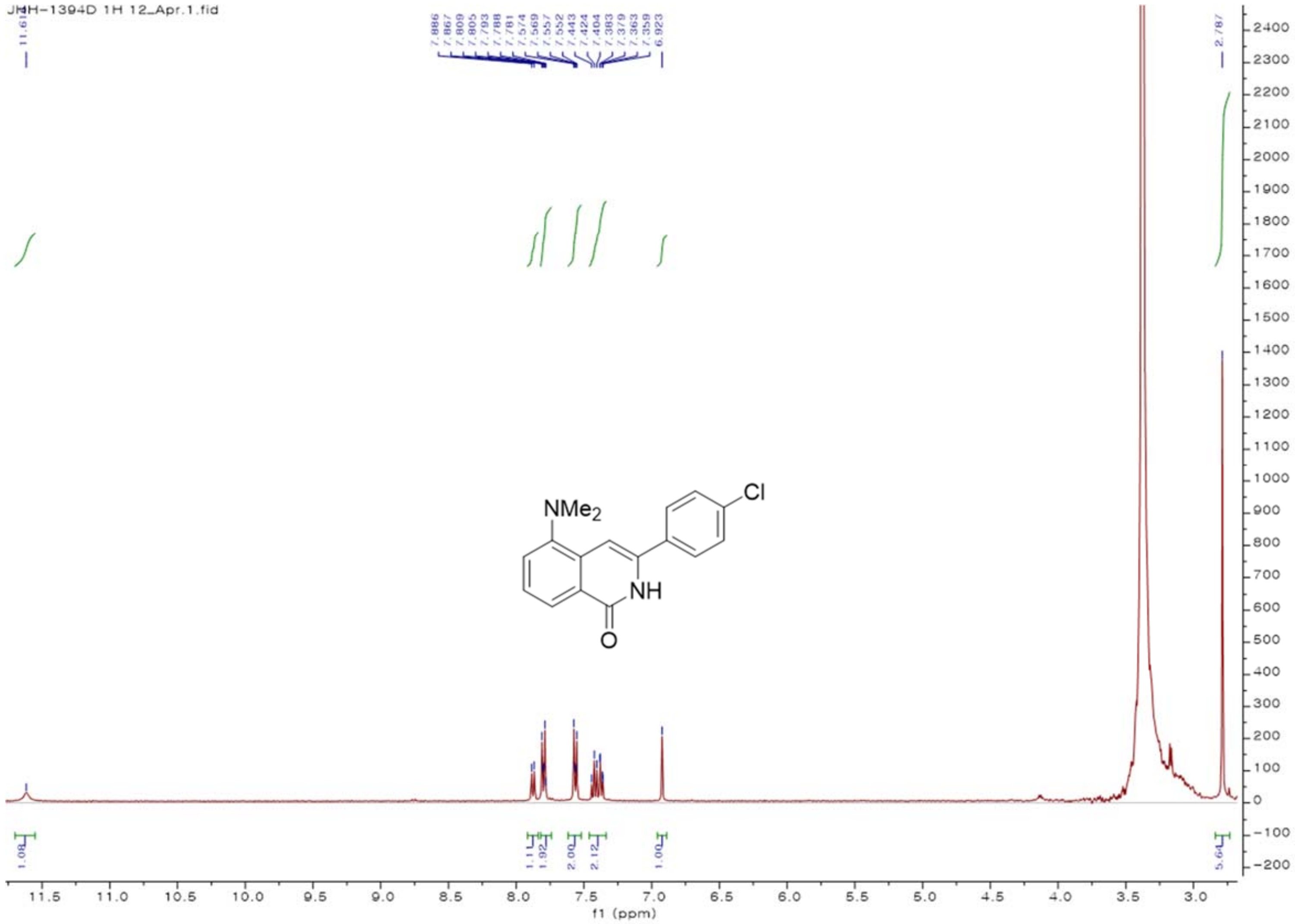

Supplementary Figure S2. ^{1}H NMR (400 MHz, DMSO-d6) spectrum of 8.

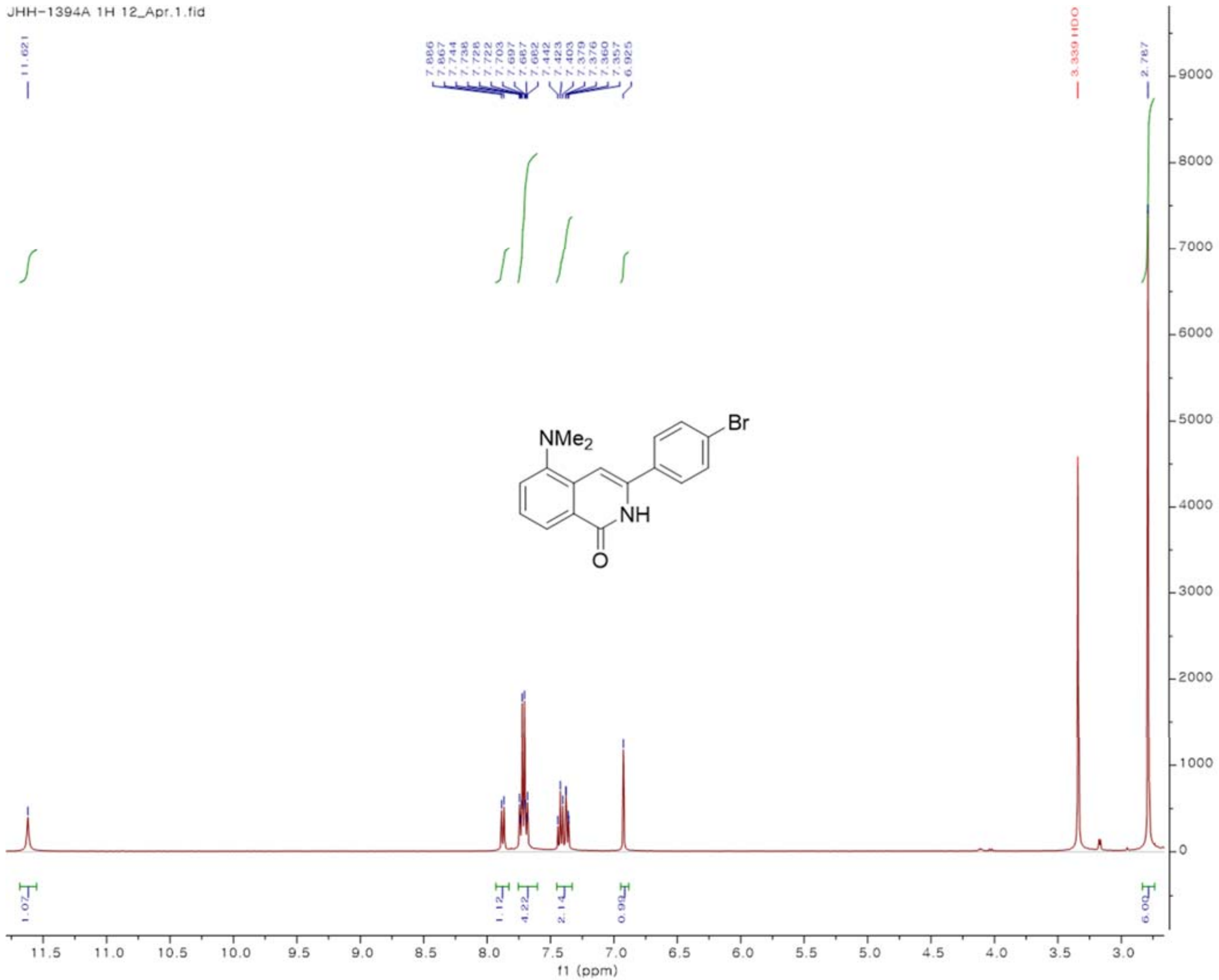

Supplementary Figure S3. ^{1}H NMR (400 MHz, DMSO-d6) spectrum of 9.

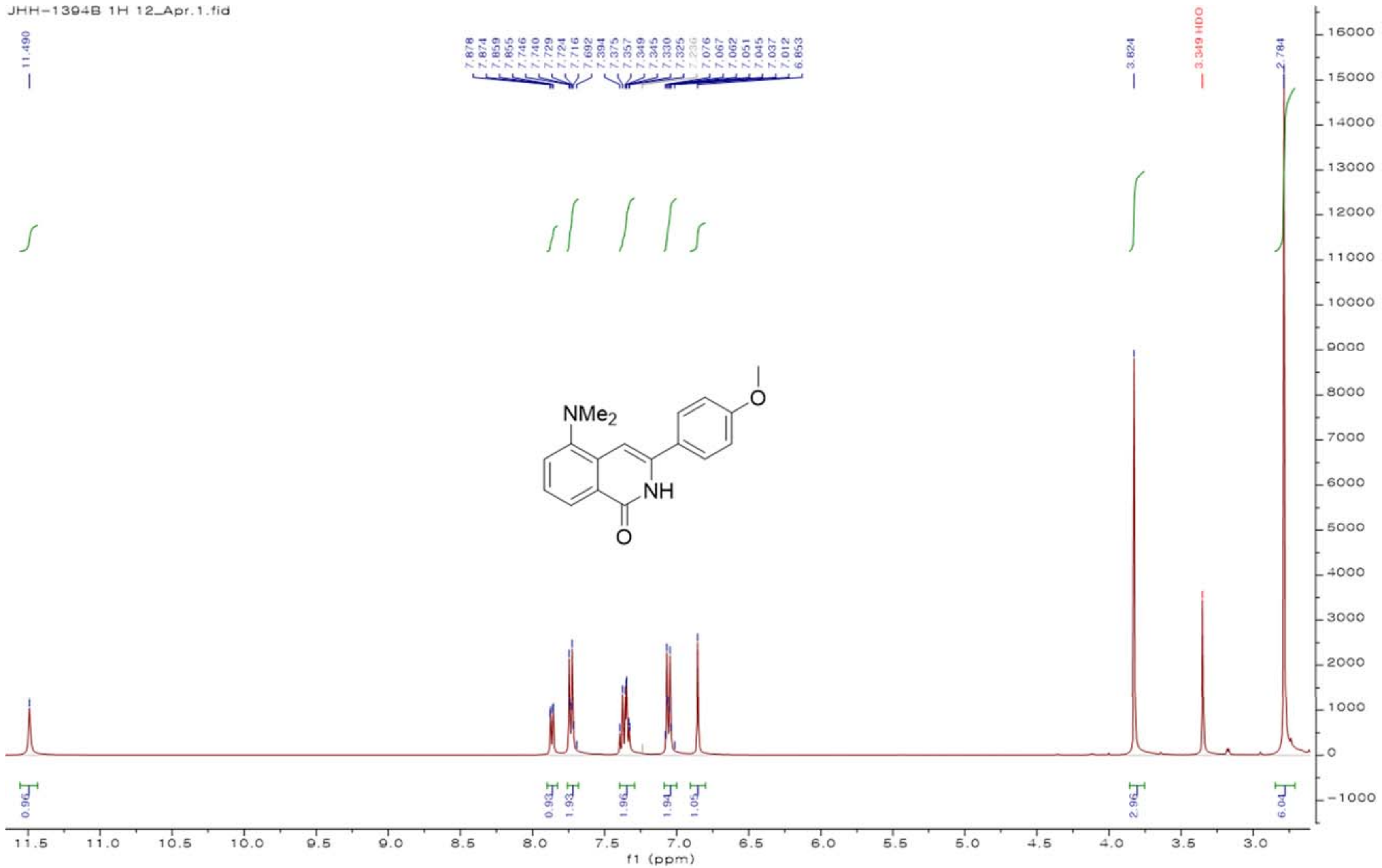

Supplementary Figure S4. ^{1}H NMR (400 MHz, DMSO-d6) spectrum of 10.

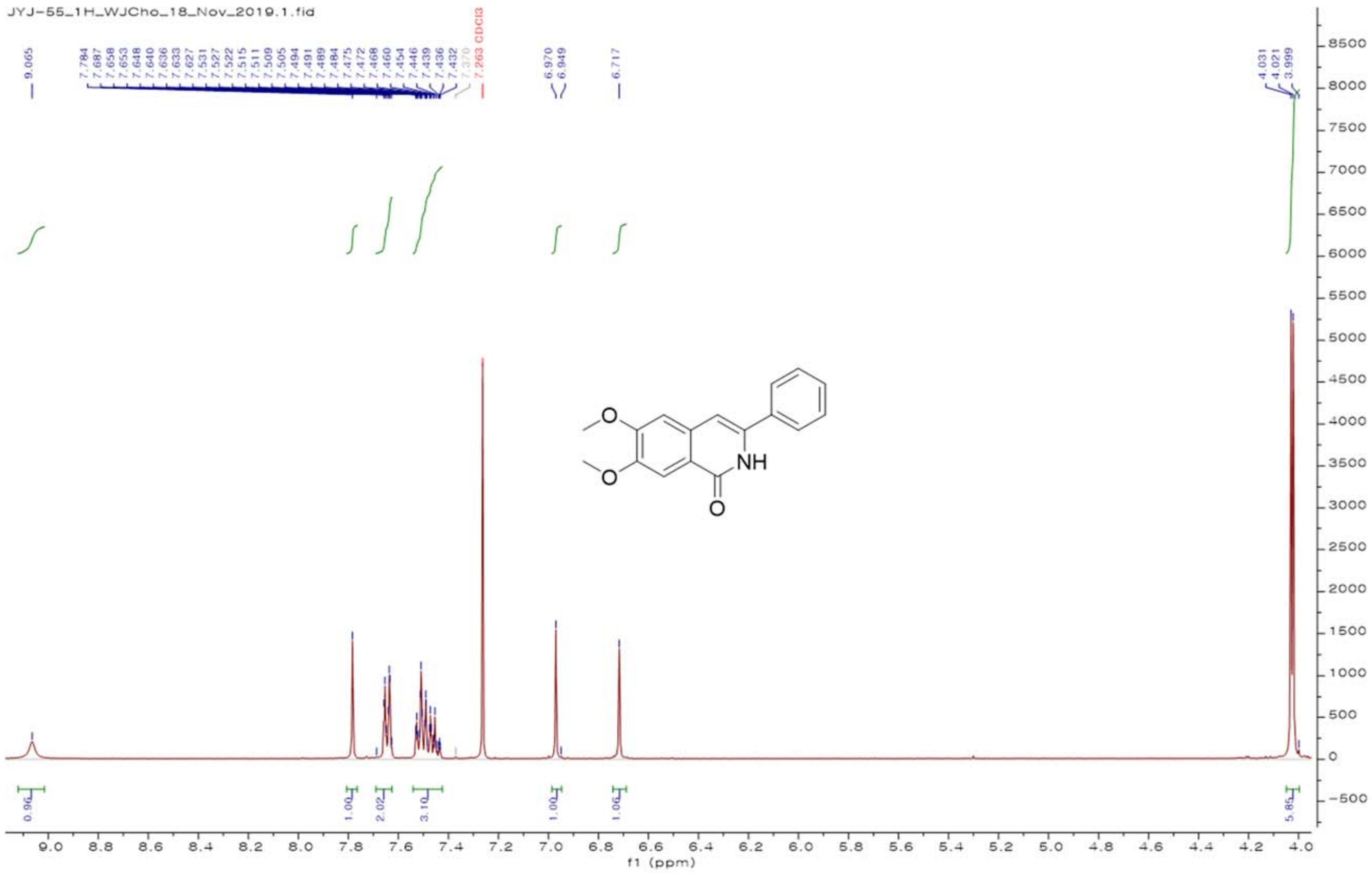

Supplementary Figure S5. ^{1}H NMR (400 MHz, DMSO-d6) spectrum of 21.

Docking Study. The software used for all docking studies was Flare versioned 3.0.0. The known x-ray 3D crystal structure of PB1 with T705, PB2 with VX-787, and PA with Baloxavir coded 4KN6, 4P1U, and 7K0W were retrieved from Protein Data Bank via software built-in function. The chemical structures of to-be-docked compound **21** were drawn by using the sketch molecule function and then minimized to their lowest energy states. For each protein, the binding site was defined based on its co-ligand. The preparation of protein was executed and the docking procedure of each inhibitor together with **21** using slow and accurate mode with 20 poses tested was carried out. After checking each poses, only the best and reasonable pose was analyzed in each case. The visualization of each results was performed in Dicovery Studio Visualizer and 2D figure was generated using its build-in fucntion.

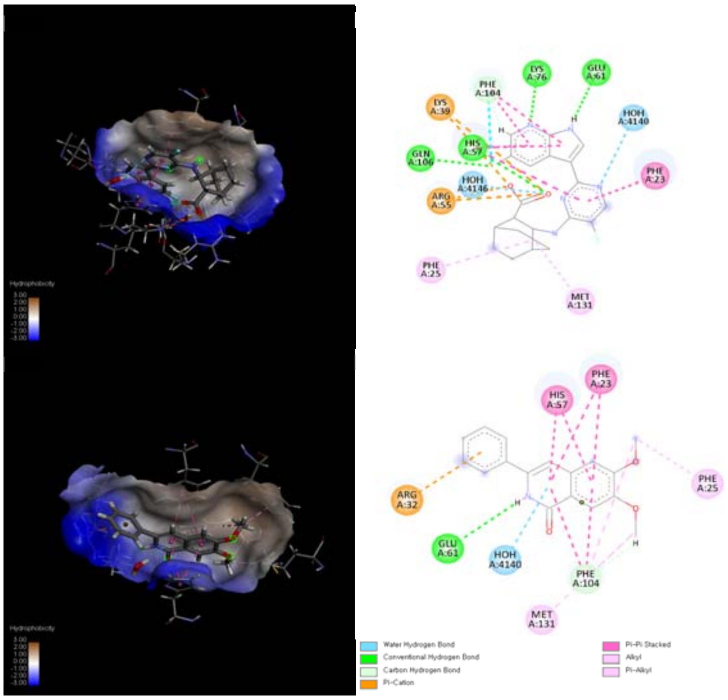

Supplementary Figure S6. Docking pattern comparison of VX787 (A and B) and compound **21** (C and D) to PB2 (PDB ID: 4P1U).

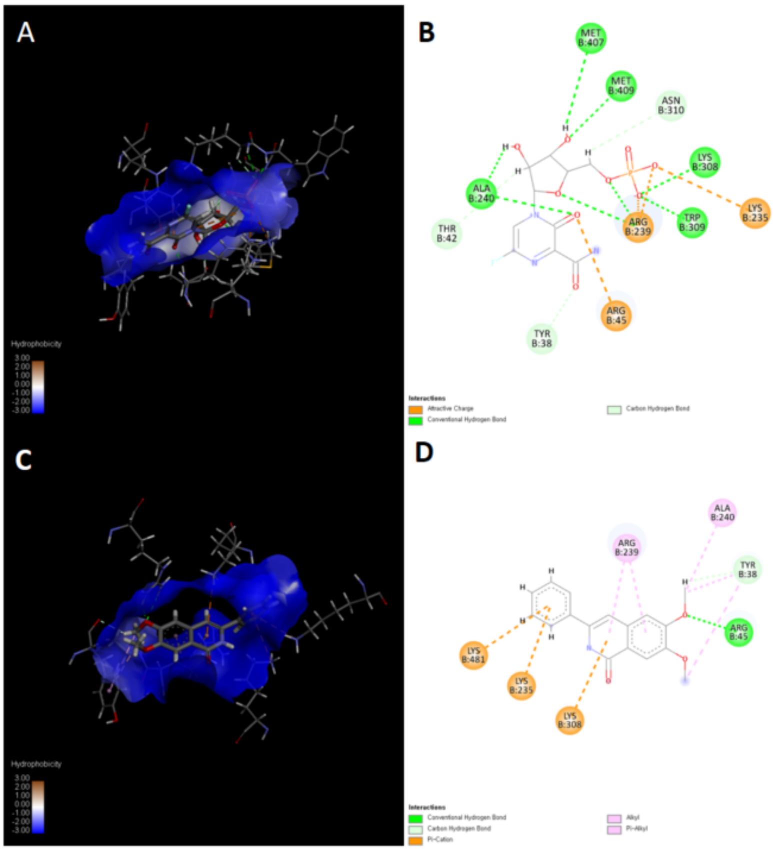

Supplementary Figure S7. Docking pattern comparison of T705 (A and B) and compound **21** (C and D) to PB1 (PDB ID: 4WSB).

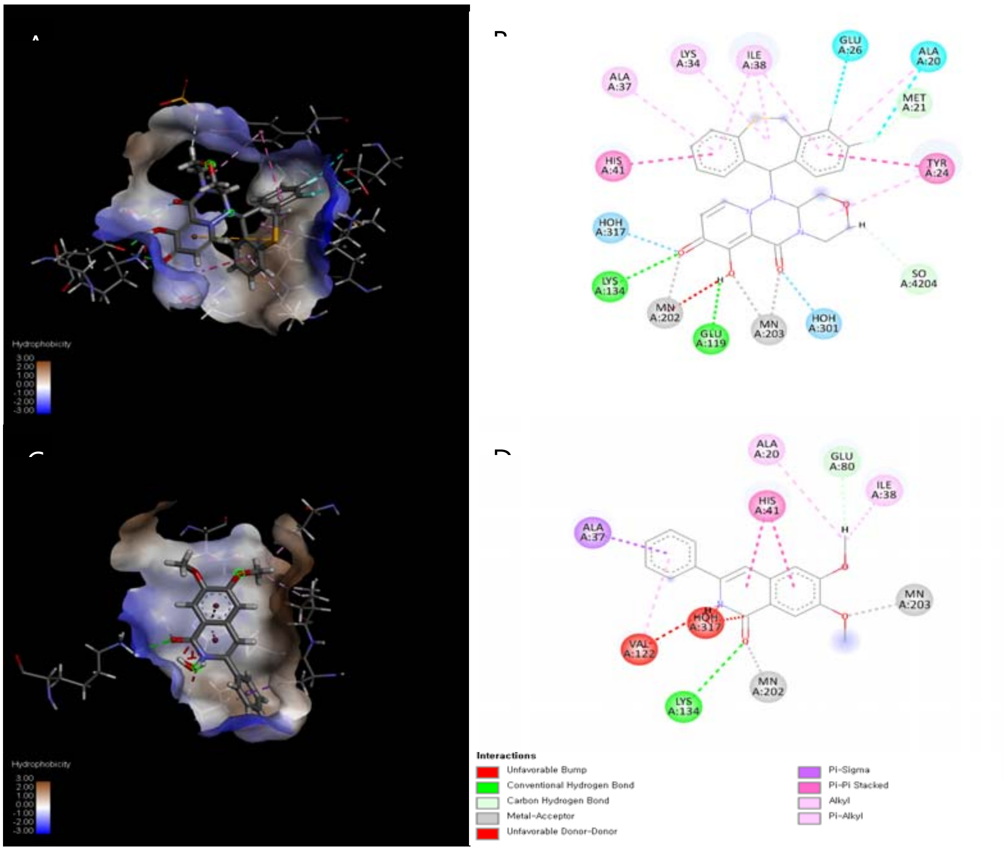

Supplementary Figure S8. Docking pattern comparison of Baloxavir (A and B) and compound **21** (C and D) to PA (PDB ID: 7K0W).
